# Acute appendicitis complicated by mass formation occurring simultaneously with serologically proven dengue fever: a case report

**DOI:** 10.1186/1752-1947-8-116

**Published:** 2014-04-03

**Authors:** Manouri P Senanayake, Malik Samarasinghe

**Affiliations:** 1 Department of Pediatrics, Faculty of Medicine, University of Colombo, Kynsey Road, Colombo, Sri Lanka; 2 Lady Ridgeway Hospital for Children, Colombo, Sri Lanka; 3 Department of Surgery, Faculty of Medicine, University of Colombo, Kynsey Road, Colombo, Sri Lanka

**Keywords:** Acute appendicitis, Dengue fever

## Abstract

**Introduction:**

Acute abdomen and acute appendicitis are unusual clinical presentations that occur in dengue infection–caused illness. Lymphoid hyperplasia and mesenteric adenitis are possible explanations, although vasculitis in the pathology of dengue infection has not been reported. Authors of previous case reports have described mimicking of acute appendicitis discovered upon surgical treatment. Dengue virus has not been proven to cause acute appendicitis.

**Case presentation:**

We report a case of an 8-year-old Sinhalese boy who developed acute appendicitis during the acute phase of serologically confirmed dengue fever. Although abdominal pain, vomiting and right-sided tenderness were present at the time of admission, a diagnosis of acute appendicitis was considered only 18 hours later, when abdominal guarding and a well-defined mass in the right iliac fossa were detected clinically and ultrasonographically. Conservative management with intravenous antibiotics was successful.

**Conclusion:**

In areas where dengue is endemic, awareness of dengue viral infection as a non-surgical cause of acute abdomen, as well as its ability to mimic acute appendicitis, is important because unnecessary surgery-related morbidity can be decreased. However, delaying or missing the diagnosis of acute appendicitis can result in serious complications. This message is particularly relevant to clinicians, especially pediatricians and surgeons, who encounter large numbers of patients during dengue epidemics and run the risk of missing the diagnosis of acute appendicitis. Likewise, delaying or missing the diagnosis of dengue hemorrhagic fever can lead to dengue shock syndrome and even death. This case highlights the need for careful evaluation of each patient who presents with acute abdomen and dengue infection.

## Introduction

Dengue infection is known to mimic acute appendicitis. The resultant diagnostic confusion has been highlighted in several case series, and dengue is now a recognized non-surgical cause of acute abdomen [1-5]. In our case report, we describe the rare occurrence of both dengue infection and acute appendicitis in a child living in a dengue-endemic area. This case highlights the need for careful evaluation, a message which is particularly relevant in dengue epidemics, when the possibility of delays in the diagnosis of acute appendicitis is high.

## Case presentation

During a dengue epidemic in Sri Lanka, an 8-year-old Sinhalese boy presented to our children’s hospital in Colombo on the fourth day of high fever that had responded poorly to paracetamol. Severe myalgia and anorexia were present from the onset of illness. Abdominal pain and vomiting had set in on the second day of symptomatic infection and abdominal pain continued for six days after hospital admission. His examination showed that he had a flushed appearance and was febrile and moderately dehydrated. His Hess test was positive. A tender hepatomegaly of 2cm and tenderness in the lower right abdomen were present. The patient’s spleen was not palpable. Laboratory tests revealed leukopenia (4×10^9^/L white blood cell count), neutrophil leukocytosis (92%), thrombocytopenia (86×109/L), raised hematocrit level (42%), and mildly elevated liver enzymes (aspartate aminotransferase, 101IU/L; alanine aminotransferase, 48IU/L). A clinical diagnosis of dengue fever was made. The boy’s dehydration was corrected with intravenous fluid therapy. The presence of immunoglobulin G (IgG) and IgM dengue antibodies provided serological confirmation of the diagnosis.

The patient was monitored for evidence of dengue hemorrhagic fever (DHF) and dengue shock syndrome. Plasma leakage and circulatory instability were absent. Eighteen hours after the boy’s admission, his abdominal tenderness became more marked. We observed localized abdominal guarding and felt a well-defined mass in the right iliac fossa. A tender mass in the right iliac fossa with surrounding intraperitoneal fluid suggestive of an appendicular mass was confirmed by ultrasonography. The patient’s serum C-reactive protein (CRP) level was 44.0mg/L (normal, <8mg/L). His urine culture was sterile. Concomitant acute appendicitis with dengue fever was diagnosed clinically and on the basis of ultrasonography.

The boy’s illness was managed conservatively with intravenous cefotaxime and metronidazole. The patient’s leukopenia and thrombocytopenia resolved on the sixth day of illness, and we observed the characteristic rash that appears upon recovery from dengue. However, his fever, neutrophil leukocytosis, and raised CRP level persisted. We attributed these symptoms to appendicitis and mass formation. We administered intravenous antibiotics, which led to a positive response, and the patient was free of pain and gastrointestinal symptoms when he was discharged to home. An interval appendectomy was planned. His hospital stay of 13 days exceeded the average length of stay for dengue fever.

## Discussion and conclusion

Dengue is an important viral infection in the world today. Lymphoid hyperplasia and mesenteric adenitis may be possible underlying pathologies producing the unusual clinical presentation of acute abdomen in dengue illness, which sometimes mimics acute appendicitis. Other possible causes of acute abdomen are the pathophysiologic changes that occur at the onset of shock in DHF or upon the simultaneous occurrence of acute appendicitis. To the best of our knowledge, vasculitis has not been reported in conjunction with dengue infection. The incidence of acute abdomen in dengue illness has been reported to be as high as 12%, which occurred during a dengue epidemic in Pakistan [6]. Five (1.69%) of the seven patients diagnosed with acute appendicitis in the report by Shamim underwent appendectomies, but histological confirmation was not mentioned [6]. It is important that clinicians in dengue-endemic areas be aware of this overlapping presentation in order to avoid unnecessary surgery-related morbidity or even mortality.

The patient we describe in this report developed acute appendicitis and dengue fever simultaneously. The development of an appendicular mass in our patient indicates a delay in diagnosis. Abdominal symptoms and signs were present upon his admission to our hospital, but they were misinterpreted as mimicry. Retrospectively, the persistence of the boy’s fever beyond the viremic phase and the occurrence of neutrophil leukocytosis and raised serum CRP were indicators of the clinical diagnosis of dengue and acute appendicitis and supported the presence of an inflammatory process.

No test to measure our patient’s erythrocyte sedimentation rate (ESR) was performed. It is a simple investigation that would have been helpful because ESR is normal in patients with DHF and lower than normal in those with shock [7], but is elevated in patients with acute appendicitis.

The case of our patient underscores the need for careful evaluation, including ultrasonography, of patients with acute abdomen, even when the diagnosis of dengue infection is confirmed. Delay in the diagnosis of dengue infection can cause dengue shock syndrome or even death. Likewise, delaying or missing the diagnosis of acute appendicitis can result in serious complications. This message is particularly pertinent during dengue seasons, when hospital admission rates to pediatric wards are very high.

## Consent

Written informed consent was obtained from the patient’s parents for publication of this case report and any accompanying images. A copy of the written consent is available for review by the Editor-in-Chief of this journal.

## Competing interests

The authors declare that they have no competing interests.

## Authors’ contributions

MPS analyzed and interpreted the patient data and was the major contributor to the writing of the manuscript. MS provided his opinion regarding and confirmation of the diagnosis from a surgeon’s point of view and agreed with the conservative management of the patient. The clinical care of the patient was shared by MS and MPS. Both authors read and approved the final manuscript.
